# Ventilatory response to hypercapnia is increased after 4 h of head down bed rest

**DOI:** 10.1038/s41598-021-81837-w

**Published:** 2021-01-25

**Authors:** K. R. Murray, S. Wasef, Heather Edgell

**Affiliations:** 1 School of Kinesiology and Health Science, York University, 355 Bethune College, 4700 Keele St, Toronto, ON M3J 1P3 Canada; 2 Muscle Health Research Centre, York University, Toronto, ON Canada

**Keywords:** Neuroscience, Physiology, Medical research

## Abstract

Head-down bed rest (HDBR) has previously been shown to alter cerebrovascular and autonomic control. Previous work found that sustained HDBR (≥ 20 days) attenuates the hypercapnic ventilatory response (HCVR); however, little is known about shorter-term effects of HDBR nor the influence of HDBR on the hypoxic ventilatory response (HVR). We investigated the effect of 4-h HDBR on HCVR and HVR and hypothesized attenuated ventilatory responses due to greater carotid and brain blood flow. Cardiorespiratory responses of young men (n = 11) and women (n = 3) to 5% CO_2_ or 10% O_2_ before and after 4-h HDBR were examined. HDBR resulted in lower HR, lower cardiac output index, lower common carotid artery flow, higher SpO_2_, and higher pulse wave velocity. After HDBR, tidal volume and ventilation responses to 5% CO_2_ were enhanced (all P < 0.05), yet no other changes in cardiorespiratory variables were evident. There was no influence of HDBR on the cardiorespiratory responses to hypoxia (all P > 0.05). Short-duration HDBR does not alter the HVR, yet enhances the HCVR, which we hypothesize is a consequence of cephalic CO_2_ accumulation from cerebral congestion.

## Introduction

As little as 4 h of head-down bed rest (HDBR) has been used to study the cardiovascular effects of simulated microgravity^1–3^. Short-duration HDBR mimics the cephalic fluid shift that occurs during exposure to microgravity^4,5^ and results in reduced indices of venous return^6^, decreased plasma volume^3,7^, and an increased sympathetic influence on heart rate^8^. Autonomic function after HDBR could therefore be influenced by changes in multiple reflexes such as the baroreflex, chemoreflexes, or their interactions. We have recently found that unloading the baroreceptors acutely during 5 min upright tilt can augment the ventilatory response to breathing 5% CO_2_^9^ suggesting that the HDBR posture (i.e. loading the baroreceptors) may attenuate the chemoreflex. Indeed, longer duration HDBR studies have found lower CO_2_ chemosensitivity after 20 or 120 days of HDBR^10,11^. These interactions could also be important to note from a clinical perspective as use of intraabdominal CO_2_ insufflations during surgery in 30–40° head-down posture (i.e., Trendelenburg position) increases arterial PCO_2_ within 1–2 h^12–14^, likely stimulating central chemoreceptors, and influencing autonomic control^15^.

Respiratory control appears to be influenced by complex interactions between the baroreflex, chemoreflex, and postural fluid shifts. Changes in carotid body blood flow may influence the function of the peripheral chemoreceptors. Indeed, carotid artery occlusion and therefore lower carotid body blood flow has been shown to increase the sensitivity of the hypoxic chemoreflex response in animals^16^. Further, reductions of blood pressure have been noted to increase aortic and carotid chemoreceptor firing in animal models^17–19^. These studies in animal models suggest that increased carotid body blood flow may decrease the sensitivity of the hypoxic chemoreflex in humans.

The primary objective of our study was to investigate the impact of 4-h HDBR on the hypercapnic ventilatory response (HCVR) and to determine if changes in brain blood flow were influential. Further, since hypercapnia induced hyperventilation increases the partial pressure of arterial oxygen, and alterations in carotid flow have been shown to alter peripheral chemoreflex sensitivity^9,16,20–22^, our secondary objective was to assess the hypoxic ventilatory response (HVR) in response to 4-h HDBR. Indeed, Prisk et al. found that 16 days of microgravity exposure attenuated the ventilatory response to hypoxia^23^. Therefore, we hypothesized that 4-h HDBR would attenuate both HCVR and HVR.

## Methods

### Ethical approval

All participants completed written informed consent, procedures were approved by the Office of Research Ethics at York University (ORE #e2018-029), and experiments were in accordance with the Declaration of Helsinki.

### Participant description

Eleven males and three females (23 ± 5 years, 170.4 ± 10.7 cm, 75.3 ± 17.1 kg) completed this study. Women were tested on day 8–11 of their menstrual cycle (i.e. late follicular phase to ensure a pre-ovulatory phase, when estrogen is present without progesterone, while avoiding menses), determined by self-report. All participants were free from previously diagnosed cardiovascular or respiratory conditions. Participants were instructed to abstain from fatty foods, alcohol, heavy exercise, smoking/vaping, and caffeine for 12 h prior to testing. They were also asked to consume a breakfast prior to arriving at the testing facility which consisted of 400 cal, 400 mg of sodium and 250 mL of water. Participants came for a single laboratory visit, which spanned approximately 7 h.

### Protocol

**Figure 1:**
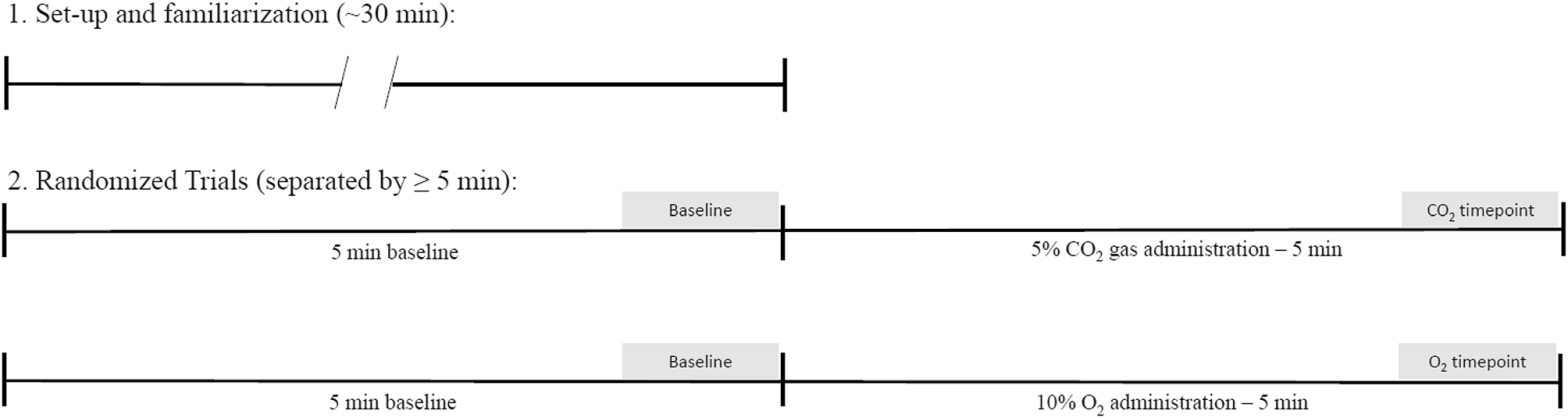
Depiction of protocol completed before and after completion of 4-h HDBR. Grey areas indicate where the data were used to calculate the Δ cardiorespiratory and cerebrovascular response to the respective gas administration.

Prior to HDBR participants completed two randomized gas mixture breathing trials to assess baseline chemoreflex function in the supine position (Fig. 1). Each trial included a 5 min baseline period of breathing room air follow by a 5 min period of breathing a gas mixture. Tests were separated by at least 5 min to ensure that cardiorespiratory data returned to baseline. Gas tank mixtures included (1) hypercapnia (5% CO_2_, 21% O_2_, balance nitrogen); and (2) hypoxia (10% O_2_, balance nitrogen) and were administered via a Douglas bag. Participants were blinded to the identity of the gas being administered, and the hypoxia test would have been terminated if arterial O_2_ saturation (SpO_2_) dropped below 80%, however, this did not occur. Participants then completed 4hrs of 6° HDBR during which they were supervised to ensure their head remained at a relative position below their feet. After HDBR, participants were briefly upright (~ 10 s) when transferring between the 6° angled bed and the supine testing bed, after which they again completed randomized gas mixture breathing tests.

### Cardiorespiratory measurements

A standard 3-lead electrocardiogram (ECG) was used to continuously measure heart rate (HR) (ADInstruments, Colorado Springs, USA). Beat-by-beat continuous blood pressure and stroke volume (SV) were measured using finger plethysmography (Nexfin, Amsterdam, Netherlands). Blood pressure was measured at the beginning of each trial using a BPTru device (Coquitlam, Canada) for subsequent offline calibration of the continuous measurements. Cardiac output (Q) was calculated as the product of HR and SV, and total peripheral resistance (TPR) was calculated as the quotient of mean arterial pressure (MAP) and Q. Changes in cardiac output as measured by the Modelflow algorithm have been shown to be correlated to changes in cardiac output as measured by Doppler echocardiography^24^. Using the Dubois and Dubois formula^25^ SV (and therefore Q and TPR) was normalized to body surface area to calculate their respective indices (Qi_,_ SVi, TPRi, respectively). A heated, linear pneumotachometer (Hans Rudolph Inc, Shawnee Mission, USA) was used to collect ventilatory data and was calibrated using a 3L syringe before each test. Tidal volume (V_T_) was calculated as the volume inspired, and respiration rate (RR) was calculated as the rate of inspiration. Ventilation (V_E_) was calculated as the product of RR and V_T_. End-tidal oxygen (ETO_2_) and carbon dioxide (ETCO_2_) were measured using O_2_ and CO_2_ analyzers (Vacumed, Ventura USA), and SpO_2_ was measured using finger pulse oximetry (Nexfin, Amsterdam, Netherlands).

### Carotid and cerebrovascular measurements

Transcranial Doppler (TCD) ultrasound (Multigon Industries, Elmsford, USA) was used to measure blood velocity through the right middle cerebral artery (MCA) using a 2-MHz probe. Cerebrovascular resistance index (CVRi) was calculated as the quotient of MAP and MCA mean velocity. Resistance index (RI) and pulsatility index (PI) were calculated as (MCA systolic velocity – MCA diastolic velocity) normalized to either MCA systolic velocity or MCA mean velocity, respectively. Longitudinal common carotid artery (CCA) diameter and blood velocity were measured using duplex ultrasonography (GE Medical Systems, Mississauga, Canada). CCA flow was calculated as the product of end-diastolic cross-sectional area and time-averaged velocity of 12 heart cycles using EchoPAC software (Version 201, GE Medical Systems, Mississauga, Canada).

### Autonomic indices

Pulse waveforms were measured simultaneously from the middle finger using the Nexfin waveform, and the ipsilateral great toe using a piezoelectric pulse transducer (ADInstruments, Colorado Springs, USA). The arrival time of both pulse waves was determined. Finger-toe pulse wave velocity (PWV) was calculated as (toe pulse arrival time – finger pulse arrival time)/(distance from suprasternal notch to toe – distance from suprasternal notch to finger). Heart rate variability (HRV; time domain and spectral analysis) was determined from the resting ECG recording before and after HDBR using a Hann (cosine-bell) data window (window overlap of 50%), and a fast Fourier transform size of 1024. A range of 0.04–0.15 Hz and 0.15–0.45 Hz was used for the low frequency (LF) and high frequency (HF) spectrums, respectively (LabChart Pro software; Version 8.1.9, ADInstruments, Colorado Springs, USA). Spontaneous cardiovagal baroreceptor sensitivity (cBRS) was measured at the same timepoints as HRV using the sequence method^26,27^.

### Data and statistical analysis

For all central hemodynamic, cerebrovascular, and respiratory variables 1 min averages at baseline and at the end of 5 min of gas mixture breathing were analyzed for both the hypercapnia and hypoxia trials. CCA flow was measured during the final minute of baseline and gas mixture breathing, and PWV was calculated during the same timepoints using at least 20 consecutive pulse waves. All continuous data were collected at 1000 Hz using a Powerlab data acquisition system (ADInstruments, Colorado Springs, USA), and accompanying LabChart Pro software.

**Figure 2:**
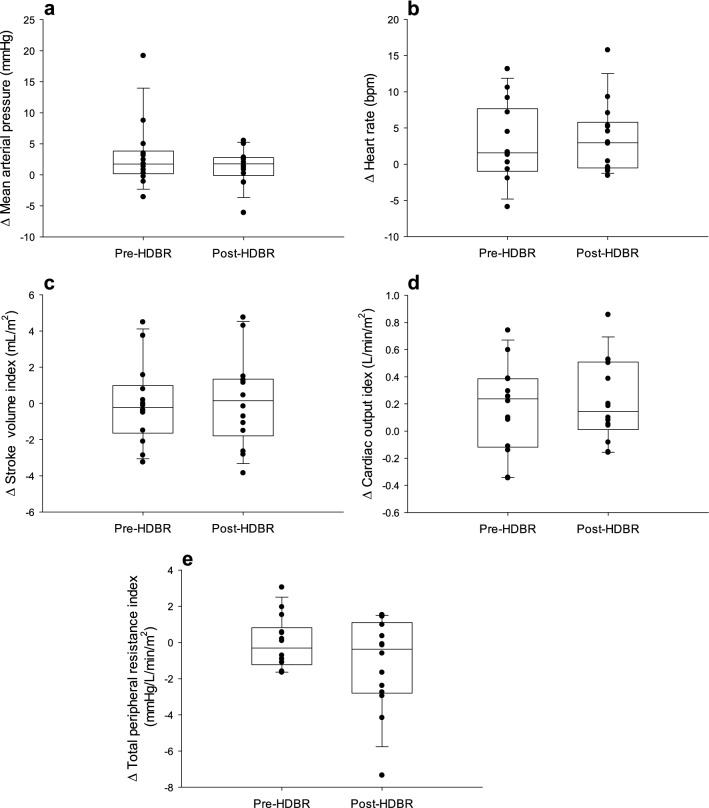
Change in mean arterial pressure **(a)**, heart rate **(b)**, stroke volume index **(c)**, cardiac index **(d)**, and total peripheral resistance index **(e)** from baseline to 5 min hypercapnia. The line within each box indicates the median, whereas the lower and upper boundaries indicate the 25th and 75th percentiles. Lower and upper whiskers represent the 10th and 90th percentiles.

**Figure 3:**
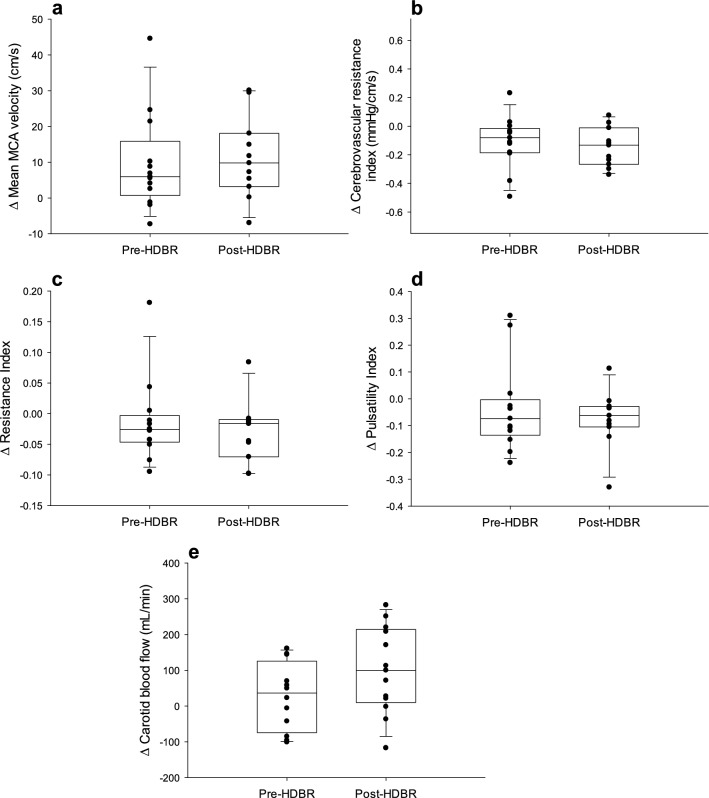
Change in middle cerebral artery mean velocity **(a)**, cerebrovascular resistance index **(b)**, resistance index **(c)**, pulsatility index **(d)**, and common carotid artery flow **(e)** from baseline to 5 min hypercapnia. The line within each box indicates the median, whereas the lower and upper boundaries indicate the 25th and 75th percentiles. Lower and upper whiskers represent the 10th and 90th percentiles.

**Figure 4:**
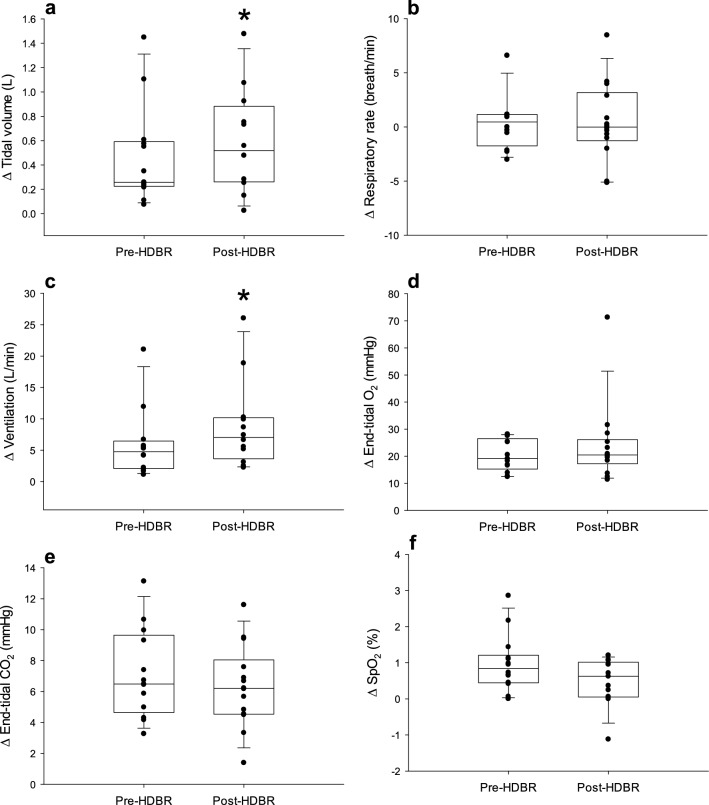
Change in tidal volume **(a)**, respiration rate **(b)**, ventilation **(c)**, end-tidal oxygen **(d)**, end-tidal carbon dioxide **(e)**, and oxygen saturation **(f)** from baseline to 5 min hypercapnia. *Significant main effect of HDBR. The line within each box indicates the median, whereas the lower and upper boundaries indicate the 25th and 75th percentiles. Lower and upper whiskers represent the 10th and 90th percentiles.

Statistical analysis was completed using Sigmaplot 13.0 (Systat Software Inc, San Jose, USA). The baseline data for cardiorespiratory, cerebrovascular, and autonomic data were obtained before and after HDBR prior to any gases were administered and were compared using a paired t-test for normally distributed data or a Wilcoxon Signed Rank Test for non-normal distributions (Tables 1 and 2). The changes in the responses to CO_2_ or O_2_ in all cardiorespiratory and cerebrovascular variables were also compared before and after HDBR using a paired t-test for normally distributed data or a Wilcoxon Signed Rank Test for non-normal distributions (Figs. 2, 3, and 4; Table 3). Gases were not compared to each other. Statistical significance was set a priori at *P* < 0.05. Data in tables are presented as mean ± standard deviation. Data in figures are presented as median with interquartile range and 10th and 90th percentile.

**Table 1:** Cardiorespiratory and cerebrovascular baseline comparisons before and after HDBR.

	Baseline	After HDBR	P-value
HR (bpm)	64 ± 9	62 ± 9*	0.03
MAP (mmHg)	81 ± 12	85 ± 9	0.19
SVi (mL/m^2^)	62 ± 7	57 ± 9	0.09
Qi (L/min/m^2^)	4.0 ± 0.7	3.5 ± 0.9*	0.03
TPRi (mmHg/L/min/m^2^)	21 ± 4	26 ± 9	0.06
MCA mean velocity (cm/s)	64 ± 10	69 ± 6	0.14
CVRi (mmHg/cm/s)	1.3 ± 0.3	1.3 ± 0.2	0.83
RI	0.59 ± 0.09	0.57 ± 0.06	0.49
PI	0.95 ± 0.23	0.89 ± 0.17	0.36
CCA flow (mL/min)	481 ± 96	412 ± 73*	< 0.001
RR (bpm)	15 ± 4	16 ± 3	0.28
V_T_ (L)	0.9 ± 0.2	0.9 ± 0.3	0.58
V_E_ (L/min)	13 ± 2	14 ± 4	0.75
ETO_2_ (mmHg)	106 ± 9	105 ± 7	0.63
ETCO_2_ (mmHg)	43 ± 5	43 ± 5	0.68
SpO_2_ (%)	98 ± 1	99 ± 1*	0.007

*HR* heart rate, *MAP* mean arterial pressure, *SVi* stroke volume index, *Qi* cardiac index, *TPRi* total peripheral resistance, *MCA* middle cerebral artery, *CVRi* cerebrovascular resistance index, *RI* resistance index, *PI* pulsatility index, *CCA *common carotid artery, *RR* respiration rate, *V*_*E*_ ventilation, *V*_*T*_ tidal volume, *ETO*_*2*_ end-tidal oxygen, *ETCO*_*2*_ end-tidal carbon dioxide, *SpO*_*2*_ arterial oxygen saturation.

*Significant main effect of HDBR. All values are mean ± SD.

**Table 2:** Heart rate variability and cardiovagal baroreceptor sensitivity before and after HDBR.

	Baseline	After HDBR	P-value
SDRR (ms)	75 ± 31	84 ± 47	0.37
RMSSD (ms)	76 ± 58	90 ± 72	0.28
pRR50 (%)	44 ± 25	49 ± 25	0.17
Total power (µs^2^)	6576 ± 6592	9793 ± 14,839	0.40
LF (nu)	31 ± 20	39 ± 23	0.12
HF (nu)	68 ± 19	60 ± 23	0.13
LF/HF	0.63 ± 0.67	0.98 ± 1.05	0.17
SD1 (ms)	54 ± 41	62.1 ± 51.7	0.28
SD2 (ms)	89 ± 26	100.0 ± 48.5	0.44
cBRS slope (ms/mmHg)	22 ± 10	25 ± 13	0.46
Finger-toe PWV (cm/s)	7.5 ± 2.3	8.4 ± 1.9*	0.03

*SDRR* SD between RR intervals, *RMSSD* square root of the mean difference between adjacent beats, *pRR50* proportion of RR interval differences greater than 50 ms divided by total beats, *LF* low frequency, *HF* high frequency, *SD1* width of the Poincaré plot, *SD2* length of the Poincaré plot, *cBRS* cardiovagal baroreceptor sensitivity, *PWV* pulse wave velocity.

*Significant effect of HDBR. All values are mean ± SD.

**Table 3:** Change in cardiorespiratory and cerebrovascular responses to hypoxia before and after HDBR.

	Baseline	After HDBR	P-value
HR (bpm)	8.7 ± 4.3	8.0 ± 4.7	0.65
MAP (mmHg)	1.5 ± 4.8	1.8 ± 3.5	0.84
SVi (mL/m^2^)	− 0.8 ± 1.8	− 0.9 ± 2.6	0.91
Qi (L/min/m^2^)	0.5 ± 0.2	0.4 ± 0.3	0.56
TPRi (mmHg/L/min/m^2^)	− 1.9 ± 1.5	− 1.6 ± 1.7	0.49
MCA mean velocity (cm/s)	− 1.2 ± 6.4	− 1.8 ± 4.1	0.42
CVRi (mmHg/cm/s)	0.07 ± 0.17	0.06 ± 0.09	1.00
RI	− 0.005 ± 0.046	0.009 ± 0.025	0.47
PI	− 0.007 ± 0.125	0.027 ± 0.072	0.52
CCA flow (mL/min)	14 ± 173	37 ± 76	0.22
RR (bpm)	− 1.0 ± 2.2	− 0.8 ± 2.6	0.96
V_T_ (L)	− 0.25 ± 0.22	0.28 ± 0.32	0.65
V_E_ (L/min)	2.8 ± 3.4	3.3 ± 5.4	0.62
ETO_2_ (mmHg)	− 48 ± 8	− 48 ± 12	0.46
ETCO_2_ (mmHg)	− 2.3 ± 2.5	− 3.2 ± 2.5	0.28
SpO_2_ (%)	− 6.1 ± 3.7	− 6.0 ± 4.2	0.86

*HR* heart rate, *MAP* mean arterial pressure, *SVi* stroke volume index, *Qi* cardiac index, *TPRi* total peripheral resistance, *MCA* middle cerebral artery, *CVRi* cerebrovascular resistance index, *RI* resistance index, *PI* pulsatility index, *CCA* common carotid artery, *RR* respiration rate, *V*_*E*_ ventilation, *V*_*T*_ tidal volume, *ETO*_*2*_ end-tidal oxygen, *ETCO*_*2*_ end-tidal carbon dioxide, *SpO*_*2*_ arterial oxygen saturation.

All values are mean ± SD.

## Results

### Baseline changes

After 4-h HDBR participants had significantly lower HR, Qi, CCA flow, and higher SpO_2_ (Table 1). There were no significant main effects of HDBR on MAP, SVi, TPRi, MCA mean velocity, CVRi, RI, PI, RR, V_T_, V_E_, ETO_2_, or ETCO_2_ (Table 1). Finger-toe PWV was significantly higher after 4-h HDBR, but there were no significant effects of 4-h HDBR on any HRV variable (SDRR, RMSSD, pRR50, total power, LF, HF, LF/HF, SD1, SD2. cBRS; Table 2).

### Hypercapnia

After 4-h HDBR, there were no significant changes in the responses of MAP (P = 0.23), HR (P = 0.19), SVi (P = 0.83), Qi (P = 0.50), or TPRi (P = 0.15) to hypercapnia (Fig. 2). Similarly, after 4-h HDBR, there were no significant changes in the responses of MCA mean velocity (P = 0.94), CVRi (P = 0.74), RI (P = 0.69), PI (P = 0.51), or CCA blood flow (P = 0.15) to hypercapnia (Fig. 3). However, after 4-h HDBR, while there was no change in the response of RR (P = 0.73), ETO_2_ (P = 0.47), ETCO_2_ (P = 0.20), or SpO_2_ (P = 0.06) to hypercapnia, there was an augmented V_T_ (P = 0.04) and V_E_ (P = 0.02) response to hypercapnia (Fig. 4).

### Hypoxia

After 4-h HDBR there were no significant changes in the responses of HR, MAP, SVi, Qi, TPRi, MCA mean velocity, CVRi, RI, PI, CCA flow, RR, V_T_, V_E_, ETO_2_, ETCO_2_, or SpO_2_ to hypoxia (Table 3).

## Discussion

After HDBR, we observed an augmented HCVR, refuting our first hypothesis of a reduced respiratory CO_2_ chemoreflex after 4-h HDBR. Second, there was no effect of HDBR on HVR suggesting no change in the respiratory O_2_ chemoreflex also refuting our second hypothesis. Since indices of cerebrovascular flow were not different after HDBR, we hypothesize that these changes in ventilatory responses (or lack thereof) could be due to exposure to greater cephalic CO_2_ concurrent with greater SpO_2_.

### Cardiorespiratory dynamics

After 20 days of HDBR, acute exposure to hypercapnia via rebreathing resulted in a reduced ventilatory response despite no change in ETCO_2_^10^. Similarly, 120 day HDBR also reduced the ventilatory response to hypercapnia^11^, which conflicts with the results of the present study. While Katayama et al. did not measure arterial CO_2_, others have documented increased arterial CO_2_ after HDBR^28^, which may be a consequence of hypoventilation from a headward shift of the diaphragm^29,30^. Others have also noted cerebral congestion^31^ and reduced cerebrospinal fluid drainage^32^ with HDBR which could potentially act in combination to increase cerebral CO_2_. Thus, the reduced HCVR documented by Katayama et al.^10^ and Vorob’yev et al.^11^ could have been due to adaptation of the CO_2_ chemoreflex^33^. In the present study, we suggest that the acute cephalic CO_2_ accumulation augmented the CO_2_ chemoreflex as adaptation had not yet occurred; however, these mechanisms remain speculative.

The effect of short-duration HDBR on HVR had not been previously examined. Given that a previous study found a significantly reduced O_2_ chemoreflex after short-duration spaceflight^23^, we expected that increased carotid chemoreceptor perfusion in the head-down posture would also reduce peripheral chemoreflex activity^16,20–22^ attenuating the ventilatory response to hypoxia; however, we observed no effect of HDBR on this response. We suggest reduced plasma volume (previously observed after 4-h HDBR)^3^ resulted in the observed reduction of Qi, which along with cerebral congestion^31^ decreased CCA blood flow. While this reduction in CCA flow might be expected to influence chemoreflex activity, we do not have evidence that flow in the carotid body itself has changed (and indices of baseline cerebrovascular resistance and ventilation are unchanged). Since the carotid body has complex autoregulatory regulation of glomus tissue to maintain flow^34,35^, we suggest this to be the case. Further, after HDBR there was a small but significant increase of SpO_2_ (likely due to better ventilation-perfusion matching) which could have contributed to lower activity of the peripheral chemoreflex.

After HDBR, the significantly reduced Qi was also driven by lower HR after HDBR. Edgell et al. (2012) found that resting plasma norepinephrine was lower after 4-h HDBR^3^ implying that plasma epinephrine may also be lower after HDBR. These reductions could help to explain the reduction of HR and Qi. MAP remained unchanged after HDBR despite lower Qi which could have been a consequence of greater regional vascular resistance (as observed with increased finger-toe PWV) and may suggest increased sympathetic activity in response to 4-h HDBR, which has been observed previously^8^. However, since there were no significant changes in HRV indices of sympathetic control of heart rate (LF:HF), and muscle sympathetic nerve activity was not measured, changes in sympathetic activity after short-duration HDBR likely require further investigation. Further, we used the Modelflow algorithm to determine SV (and thus Q and TPR) which may have increased variability in the absolute measurements. However, Modelflow measurements of Q have been shown to be highly correlated to ultrasound measurements when determining the differences between an intervention and baseline measures^24^.

### Cerebrovascular dynamics

Multiple studies have assessed the effect of HDBR on cerebral hemodynamics, but results are inconsistent and likely depend on the duration. In men, cerebral blood flow, directly measured using MRI, was reported to decrease after ~ 5-h HDBR; however, the authors also reported lower ETCO_2_ which may have confounded the effect of HDBR on brain blood flow^31^. Alternatively, studies in men for longer periods of HDBR (24-48hrs) have reported increased MCA mean velocity^36,37^, whereas no change has been reported after 2 weeks of HDBR^38^, unfortunately these studies did not report ETCO_2_. In the present study, while CCA flow was lower after 4-h HDBR, MCA mean velocity and all of the cerebrovascular indices were not significantly different which was likely due to unchanged ETCO_2_ and appropriate autoregulation.

As arterial O_2_ decreases, cerebral blood flow increases, via cerebral vasodilation, to maintain cerebral O_2_ delivery^39^. However, in the current hypoxia trial, despite decreased ETO_2_ and SpO_2_, as well as increased Qi, MCA mean velocity did not increase. This was likely the consequence of hyperventilation decreasing ETCO_2_ counteracting the expected cerebral vasodilation. Unchanged cerebrovascular indices likely indicate minimal cerebral vasomotion or even slight vasoconstriction during hypoxia in the present study. In response to hypercapnia we observed a characteristic increase in MCA mean velocity, with reductions of CVRi, RI, and PI suggesting cerebral vasodilation, but HDBR did not affect the cerebrovascular responses. To the best of our knowledge no other studies have assessed the cerebrovascular CO_2_ reactivity response after 4hrs of 6° HDBR; however, acute and severe head-down tilt (45° and 90° for ~ 20 min) does not alter MCA mean velocity, CVRi, or cerebrovascular CO_2_ reactivity either^40^. Together, these results suggest that the myogenic response of cerebral vascular smooth muscle to CO_2_ is preserved during short-duration head-down posture.

### Limitations

Firstly, only three women participated in and completed our study preventing sex-based comparisons, but previous work has noted similar hemodynamic responses between men and women after 4-h HDBR^3^. Similarly, the cerebral hemodynamic and ventilatory response to supine hypercapnia^9^, and the ventilatory response to hypoxia^41^ are not different between men and women. Secondly, since no sedentary upright control group was utilized in this study, previously observed variations in chemoreflex activation due to circadian rhythm^42,43^ could potentially confound our results. However, when specifically observing the hours of 8 am-4 pm relevant to this study, HCVR appeared similar^42,43^, and central hemodynamics have been shown to be a function of head-down posture, rather than 4hrs of inactivity, physical confinement, or time of day^3^. Thirdly, participants were upright for approximately 10 s when transitioning between the angled and supine beds for testing. While we assumed that the effects of 4-h HDBR would persist after this very brief transition, it is possible that even brief orthostasis could have affected our results. There are conflicting studies which suggest that 5 min or more of upright posture either does^44^, or does not^45^, influence the CO_2_ respiratory chemoreflex. However, we did not expect to see persistent effects of the brief upright posture as the cardiorespiratory responses to tilt recover within 5 min of the return to supine posture^46^ and our participants were supine for longer periods in preparation for testing (i.e. electrode and TCD placement, Nexfin activation, pneumotach placement, etc.). Further, after HDBR we still observed an increase of SpO_2_ which persisted past the brief orthostasis. If the orthostatic stress were to have persistently and negatively influenced the ventilation-perfusion matching in the lung, we expect that the SpO_2_ would have lowered back to pre-HDBR levels. Lastly, during hypoxic breathing our participants only had a 6% drop of SpO_2_ before and after HDBR which was a smaller drop than expected. As our participants were primarily young and healthy kinesiology students, we suggest that a larger than average blood volume could contribute to this and therefore a plateau at the SpO_2_ nadir may not have been reached after only 5 min of hypoxia. We suggest that future studies employ a longer period of exposure.

We investigated flow within the CCA rather than the internal carotid artery. The diameter of the internal carotid artery is often difficult to image, and therefore we chose to use the common carotid. While not ideal, this measurement along with the cerebrovascular resistance indices from the TCD measurements, allowed for indices of brain blood flow. Additionally, TCD ultrasound does not allow for estimation of blood vessel diameter, yet MCA velocity is often used as an index of cerebral blood flow. The impact of HDBR on MCA diameter has not been studied, but previous work using similar levels of hypercapnia has shown that CO_2_ causes vasodilation of cerebral arteries^47–49^. Therefore, the actual increase in cerebral blood flow in response to hypercapnia was likely larger than the observed increase in MCA velocity. We also observed a discrepancy between arterial oxygen saturation (higher) and ETO_2_ (unchanged) after HDBR, but this was potentially due to greater variability in ETO_2_ measurements. Future work should also utilize near-infrared spectroscopy (NIRS) or transcutaneous CO_2_ monitoring on the head to help characterize potential alterations of cerebral O_2_ and CO_2_ after 4-h HDBR. Lastly, the results of the present study cannot be generalized to older adult or clinical population as comorbidities and pathologies would likely impact the responses measured.

## Conclusions

Short-duration HDBR resulted in enhanced HCVR, yet no change in HVR. We suggest that these changes could be due to exposure to cephalic CO_2_ accumulation and higher peripheral oxygenation while in the head-down posture, respectively. We did not find evidence of changes in cerebrovascular resistance after short-term HDBR; however, Qi was lower due to reductions of HR and potential increases of vascular resistance. This enhancement of HCVR in the HDBR position could potentially influence cardiorespiratory function during cephalic fluid-shifts.

## Data Availability

The data that support the findings of this study are available from the corresponding author upon reasonable request.
